# Genotype-specific Digital Twins for Arrhythmia Ablation Targeting in Arrhythmogenic Right Ventricular Cardiomyopathy

**DOI:** 10.21203/rs.3.rs-8006874/v1

**Published:** 2025-11-05

**Authors:** Yingnan Zhang, Adityo Prakosa, Kelly Zhang, Richard Carrick, Jonathan Chrispin, Stefan L Zimmerman, Konstantinos Aronis, Eugene G. Kholmovski, Crystal Tichnell, Brittney Murray, Cynthia James, Hugh Calkins, Natalia Trayanova

**Affiliations:** 1 Department of Biomedical Engineering, Johns Hopkins University; Baltimore, MD, USA.; 2 Alliance for Cardiovascular Diagnostic and Treatment Innovation, Johns Hopkins University; Baltimore, MD, USA.; 3 Division of Cardiology, Department of Medicine, Johns Hopkins Hospital; Baltimore, MD, USA.; 4 Department of Radiology and Radiological Science, Johns Hopkins Hospital; Baltimore, MD, USA

## Abstract

Arrhythmogenic Right Ventricular Cardiomyopathy (ARVC) is a severe genetic heart disease that predominantly affects young athletic individuals and carries a high risk of ventricular tachycardia (VT) and sudden cardiac death. Although catheter-based radiofrequency ablation is the state-of-the-art treatment for VT, its success in ARVC is limited by high recurrence rates. Here, we present Genotype-specific Digital-twin Ablation Targeting (**Gen**DIRECT), a personalized, non-invasive technology that predicts, prior to the clinical procedure, the optimal VT ablation targets using patient-specific heart digital twins. The **Gen**DIRECT-predicted target set is devised to eliminate, with minimum lesion size, the ability of the ARVC ventricles to sustain any reentrant arrhythmias. Comparison of **Gen**DIRECT target lesions with the clinical ablation lesions demonstrated excellent co-localization in both ARVC patients who had undergone index ablation and in those with redo procedures many months later, regardless of genotype. Thus, should **Gen**DIRECT be used to guide clinical procedures, it is expected that existing VTs will be terminated, and post-ablation arrhythmias will be prevented, thereby increasing the efficacy of the ablation procedure and reducing redo procedures and re-hospitalization.

## Introduction

Arrhythmogenic right ventricular cardiomyopathy (ARVC) is a severe genetic heart muscle disorder characterized by life-threatening ventricular tachycardia (VT) and a high risk of sudden cardiac death (SCD)^1, 2, 3, 4^. This condition primarily affects young and active individuals; population studies have shown that ARVC accounts for 20% of SCD in athletes younger than 35 years old and 30% of sudden death in young adults^5, 6, 7^. Approximately 60% of ARVC cases are due to mutations in desmosomal genes, with plakophilin-2 (*PKP2*) being the most prevalent; about 30% are considered gene-elusive (GE), with the causative genetic variants remaining unidentified^8, 9, 10, 11, 12^. The arrhythmogenic mechanisms of *PKP2* and GE-associated ARVC cases are different, but both present with arrhythmogenic fibrotic replacement in the right ventricular (RV) myocardium^13, 14, 15, 16^. Additionally, electrophysiological (EP) remodeling from *PKP2*-related ion channel dysfunction can further disrupt electrical propagation and lead to the formation of complex reentrant arrhythmia circuits^17, 18^. The distinct arrhythmogenic substrates of *PKP2* and GE suggest potential differences in ARVC clinical presentation and a need for genotype-specific clinical management.

Catheter-based radiofrequency ablation is a key treatment for controlling VT in patients with ARVC. The procedure aims to identify critical sites of the VT reentrant circuit and render them non-conductive, thus interrupting the circuit^19^. However, the effectiveness of catheter ablation varies considerably among patients and is accompanied by a high VT recurrence rate^20, 21, 22^. Patients often require multiple repeated procedures, sometimes within short periods of time. This primarily results from challenges in identifying all possible VTs and selecting the effective ablation targets during the procedure^23, 24^. Technical limitations, such as inadequate mapping resolution and the 3D nature of the reentrant circuit, hamper the precise localization of VT-sustaining sites. Additionally, patients often exhibit hemodynamic intolerance^25, 26^ which prevents the VTs from being mapped, making it difficult to localize critical sites. These limitations can result in VT incomplete elimination, requiring additional ablations that inflict unnecessarily large lesions and compromise cardiac function, and prolonged procedures that increase complication risks^27, 28, 29^. There is, therefore, an urgent need to address these clinical challenges to enhance the therapeutic efficacy of ARVC ablation.

Digital twining is an emerging technology in precision medicine that uses a set of virtual information constructs to mimic the behavior of an organ or even a patient and has predictive capability that informs clinical decisions. Heart digital twins (DTs), which integrate the patient’s cardiac imaging, clinical information, and basic biophysical knowledge, have already made significant contributions to patient disease prognostication and therapy delivery guidance. The technology has demonstrated accurate prediction of personalized risk of sudden cardiac death by evaluating the arrhythmogenic propensity of the patient’s disease-induced substrate^30, 31, 32^. It has shown promise in non-invasively guiding the catheter ablation procedure in patients with persistent atrial fibrillation while minimizing ablation lesions^33, 34^. Beyond these clinical applications, the DT approach has also been used to dissect the arrhythmogenic mechanisms of various substrates, including fibrosis and penetrating adiposity.^35, 36, 37^.

Here, we present a genotype-specific DT ablation target prediction technology (**Gen**otype-specific DIgital-twin ablation taRgEt PrediCTion, or **Gen**DIRECT) for ARVC patients, where optimal ablation targets are determined non-invasively via personalized DT simulations. The DTs are reconstructed from each ARVC patient’s late gadolinium enhancement magnetic resonance imaging (LGE-MRI) scans and their genetic profiles; the ablation target prediction is thus custom-tailored to each patient. The efficacy of ablating the predicted optimal targets is then tested in the patient’s DT – thus the patient’s DT “is treated” first, before treating the patient, which enables comprehensive pre-procedural planning. These three-dimensional (3D) targets allow for proper guidance for epicardial and/or endocardial approach and can then be readily utilized in the clinical procedure to steer the patient’s treatment.

As **Gen**DIRECT provides the targets prior to the clinical procedure, it avoids the lengthy invasive mapping of the ARVC patient’s electrical activity to determine the VT critical sites (i.e. the clinical targets for ablation). Importantly, the approach also includes a concept that is unique to the DT technology: the set of 3D personalized targets is designed to completely eliminate the ability of the ARVC ventricles to sustain any arrhythmias. This ablation concept is radically different from any existing ablation strategies, as the goal here is to not only effectively terminate the clinically-manifested VTs, but to also prevent new arrhythmias arising after (and potentially because of) the ablation procedure, thus eliminating the need for redo ablations and re-hospitalization. Here, we demonstrate the predictive capabilities of **Gen**DIRECT in eliminating the arrhythmogenic propensity in ARVC patients in a personalized genotype-specific manner, by comparing, in a blinded fashion, **Gen**DIRECT predictions with clinical data, both on index (first) ablation and on any redo procedures. Ascertaining the predictive capabilities of **Gen**DIRECT can pave the way for a prospective randomized trial, ultimately changing the paradigm for delivering arrhythmia treatment to ARVC patients.

## Results

### Study overview

**Fig. 1:**
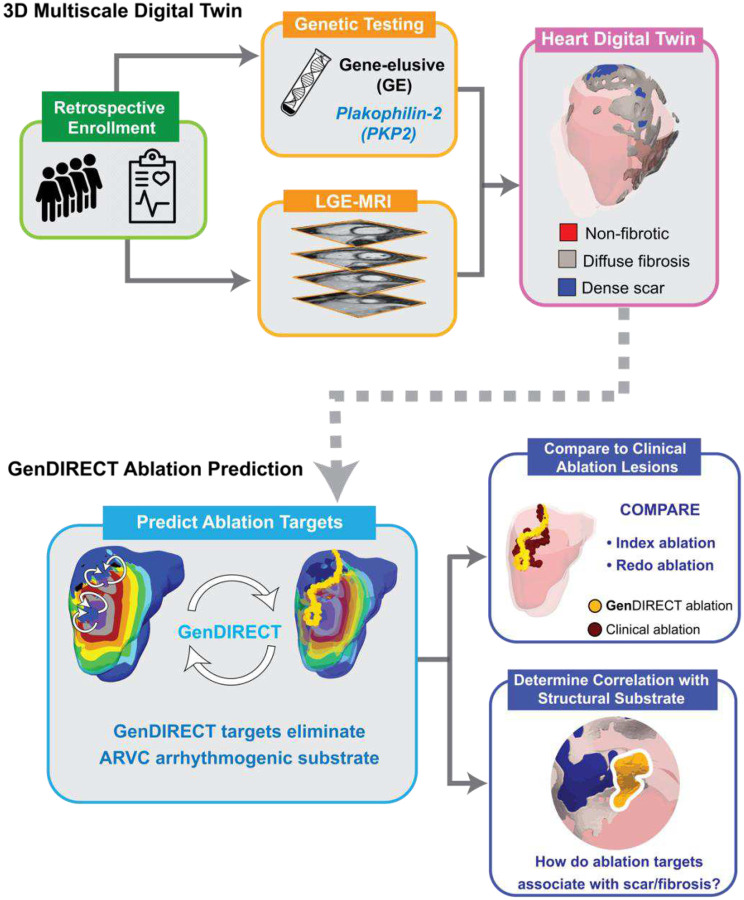
Overview of the GenDIRECT study.

In this retrospective clinical and digital-twinning study, patients diagnosed with definite ARVC^38^ who had a history of VT ablation(s) were selected from the Johns Hopkins ARVC Registry and retrospectively enrolled. For each patient, a 3D multiscale (i.e. from cell to organ) personalized DT was reconstructed using the LGE-MRI scan and the genetic testing result. Each DT thus incorporates the unique heart geometry, patient-specific distribution of structural remodeling (scar/fibrosis) and genotypic EP properties. The **Gen**DIRECT technology predicts custom-tailored ablation targets for each patient by pinpointing critical elements of the VT circuits induced in the patient’s DT by rapid pacing. The predicted targets were then ablated virtually in the DT, and VT inducibility tests were repeated to determine whether new VTs emerge post-ablation, which were, in turn, targeted in the patient’s DT, until a final set of lesions was established that eliminated completely the substrate arrhythmogenesis. To assess the predictive capability of the approach, unblinded **Gen**DIRECT predicted lesions were compared with the clinical lesions for patients that had undergone index (first) ablations of inducible VTs as well as those with redo ablations after VT recurred. Finally, the correlation between ablation targets and scar/fibrosis distributions was examined to determine whether the latter could guide substrate ablation for patients in whom VT cannot be induced as they are hemodynamically unstable. The contribution of genotypic differences in ARVC patients (*PKP2* or GE) to this correlation was also established. Figure 1 presents an overview of the study.

In the ARVC cohort enrolled retrospectively, LGE-MRI scans and genetic testing results were used to construct 3D personalized DTs. Each DT incorporates patient-specific distribution of structural remodeling and genotypic EP properties. **Gen**DIRECT predicts custom-tailored ablation targets for each patient based on the VT reentrant circuits induced in the patient’s DT. The final set of **Gen**DIRECT targets that eliminates substrate arrhythmia propensity in each DT was unblinded and compared to the clinical index ablation lesions for that patient. For patients who had repeat procedures, **Gen**DIRECT ablation targets were compared to both index and redo clinical lesions. Finally, the correlation between ablation targets and scar/fibrosis distribution was analyzed and compared between the two ARVC genotype groups, GE and *PKP2*.

### Patient demographics

This retrospective study included 30 patients diagnosed with definite ARVC. Genetic testing identified *PKP2* loss-of-function variants in 15 patients, while the remaining 15 were categorized as GE. LGE-MRI scans confirmed right ventricular (RV) hyperenhancement in all patients. No hyperenhancement on the left ventricle (LV) was observed within the cohort. Eleven out of 30 patients had an implanted cardioverter-defibrillator (ICD) implanted before the LGE-MRI. Among the patients, 25 underwent a single (index) ablation, while 5 required redo ablation(s) after their initial ablation failed and VT recurred. All redo procedures were performed within 12 months of the index ablations, with an average time interval of 7.5 months. The shortest interval was one month. Patient characteristics are summarized in Table 2.

### Clinical EAM and ablation procedure

Clinical EAM was performed for all 30 patients. The mean EAM surface area for the cohort was 406.73 ± 191.12 cm^2^, and the mean mapping density was 88 points/cm^2^. An overview of clinical ablation techniques used for all patients is provided in Table 3. Procedural VT inducibility was positive in all 30 patients (100%). Pace mapping was performed in 28 of 30 patients (93.3%). In addition to ablation guided by pace maps, 27 of 30 patients (90.0%) underwent substrate-based ablation. Of these 27 patients receiving substrate-based ablation, 25 (92.6%) were treated for fractionated or late potentials, while the remaining 2 (7.4%) were treated for slow conduction zones.

Among the 25 patients who remained free of VT recurrence after a single index ablation, all 25 (100%) underwent epicardial mapping, and 23 (92.0%) underwent endocardial mapping. Epicardial ablation was performed in 19 of these 25 patients (76.0%), and endocardial ablation in 10 (40.0%). For the 5 patients who experienced VT recurrence after their index procedure, details of their procedures are as follows. During the index ablation, all 5 patients (100%) underwent epicardial mapping and ablation, and 3 (60.0%) underwent endocardial mapping and ablation. In their subsequent redo procedures, 3 of these 5 patients (60.0%) underwent epicardial mapping and ablation, and 4 (80.0%) underwent endocardial mapping and ablation.

### GenDIRECT-predicted lesions effectively mitigate arrhythmogenicity in DTs of ARVC patients undergoing clinical index ablation

A personalized heart DT was reconstructed for each ARVC patient, incorporating patient-specific distributions of structural remodeling (scar/fibrosis derived from LGE-MRI). The patient genotype was used in a cell-level ionic model to represent the cellular EP properties, and each DT was constructed by populating the imaging-based geometric model of the patient’s heart with the genotype-specific cell model, as described in the Methods. Following a rapid pacing from 9 locations on the RV endocardial surface in the patient’s DT that served as a surrogate for potential arrhythmia triggers, all induced VTs were examined. For each DT, **Gen**DIRECT predicted, blind to the clinical ablation, custom-tailored ablation targets by pinpointing the critical sites for all VT reentrant circuits induced in the DT. Following digital ablation, VT inducibility tests were repeated in each DT to assess the arrhythmogenic propensity of the new structural substrate, which comprised the original fibrosis/scar distribution and the new ablation lesions. If new VTs emerged, they were targeted by **Gen**DIRECT and this process was repeated until VT non-inducibility of the DT was achieved.

**Fig. 2.**
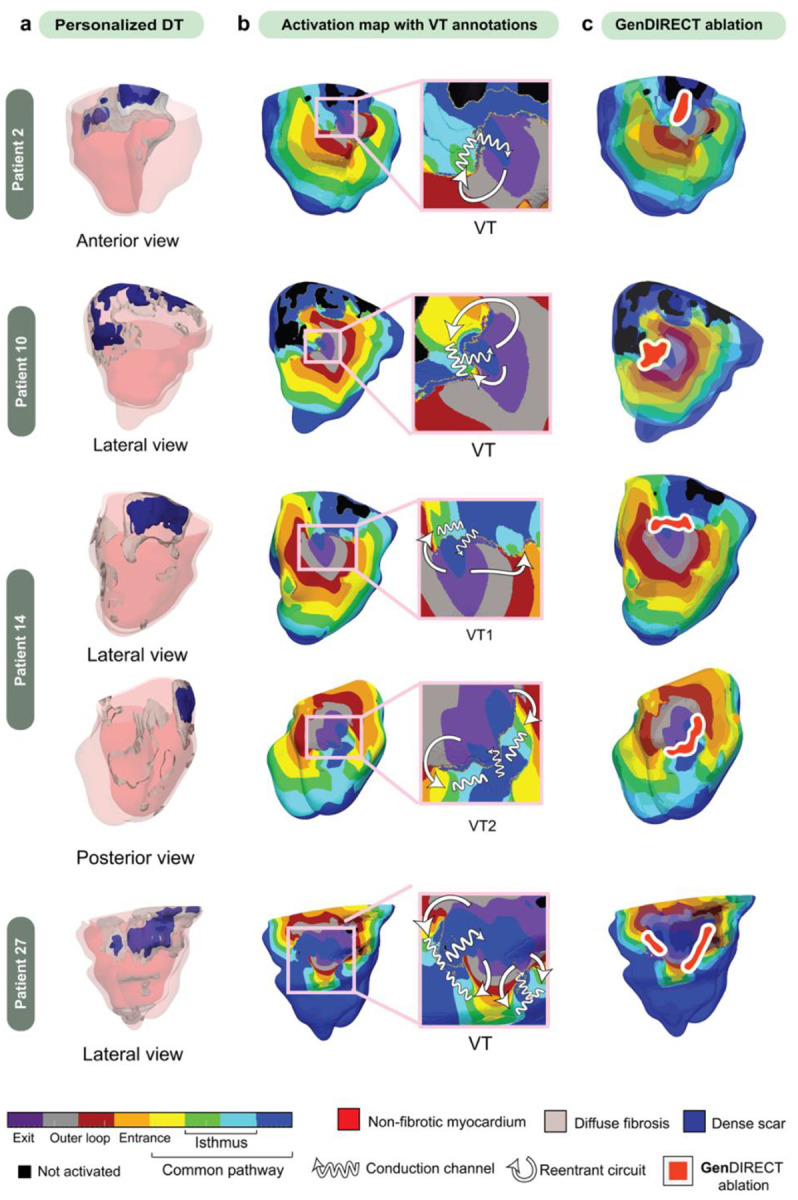
Personalized DTs and VT activation maps with annotations for four ARVC patient cases. **a** Distribution of disease-remodeled tissue in the DTs, with diffuse fibrosis in gray and dense scar in dark blue. **b** VT activation maps with critical components of the VT circuit annotated. The activation maps were color-scaled into eight segments, namely exit (purple), outer loop (gray and red), entrance (orange), isthmus (green and cyan) and common pathway (yellow to dark blue). White curved arrows trace the VT circuit from the exit site to the common pathway and wiggly arrows represent VT isthmuses. **c Gen**DIRECT ablation targets (filled-in orange with white contour) of these four patients. These targets render the DTs non-inducible for VT.

A total of 270 DT simulations were conducted across the 30 ARVC patients. Sustained VT circuits were successfully induced in all DTs. These simulations generated high-resolution VT activation maps, revealing 57 unique VT morphologies in total—29 in the GE group and 28 in the *PKP2* group. Fig. 2 depicts four representative DTs, with patients 2 and 10 being GE and patients 14 and 27 being *PKP2*, and the corresponding predicted ablation targets. For each DT, all the induced unique VT reentrant circuits were annotated following established methodologies used in clinical EAM data annotation to identify key circuit components: exit, entrance, outer loop, isthmus, and common pathway^39^. The VT isthmuses, which are the critical slow-conducting narrow pathways that sustain reentry and are the primary targets for clinical ablation, are highlighted in Fig. 2b with wiggly arrows. Subsequently, as shown in Fig. 2c, **Gen**DIRECT ablations (orange with white contour) were performed for all VT isthmuses in each of the DTs. Arrhythmogenic propensity was successfully eliminated in all the DTs, rendering them non-inducible following a repeated rapid pacing protocol.

### Comparison between GenDIRECT predicted lesions and clinical ablation lesions at index VT ablations

**Fig. 3:**
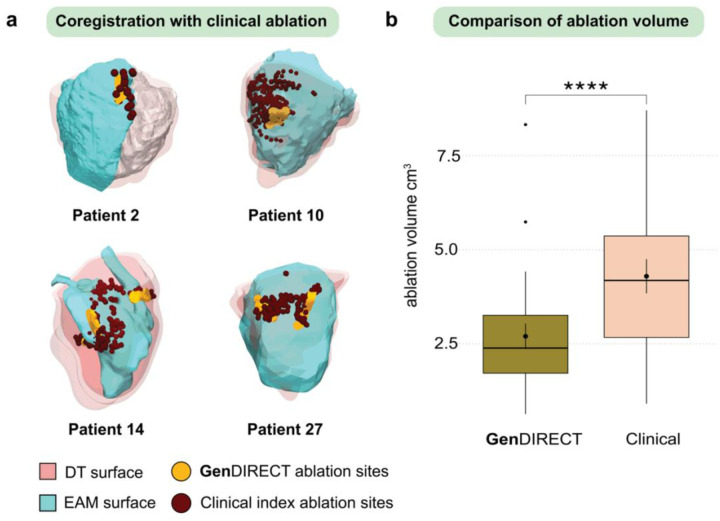
Comparison of GenDIRECT ablation targets and clinical index ablation lesions. **a** Examples of the same four patients from Fig. 2, each having undergone a single clinical index ablation. Co-registered EAM (cyan) and DT (light pink) surfaces are shown. Clinical index ablation lesions (dark red) and **Gen**DIRECT targets (orange) were projected onto the same endocardial surface of the DT for direct comparison. A Dice score was computed for each patient’s case to quantify the overlap between **Gen**DIRECT and clinical ablation targets. **b** For patients undergoing only one clinical index ablation procedure, the volume of **Gen**DIRECT ablation targets (olive, 2.70 ± 1.69 cm3) was significantly smaller than the volume of clinical index ablation lesions (nude, 4.29 ± 2.27 cm3). Wilcoxon signed rank test was used to compare between the two paired groups and assess statistical significance. *N* = 25, ****p < 0.0001.

For the 25 patients who underwent a single successful clinical index ablation (i.e first ablation) with VT not recurring, the **Gen**DIRECT-identified ablation sites were compared to the unblinded clinical index ablation lesions derived from intra-procedural EAM data (see Methods for detailed description). Figure 3a provides examples of this comparison using the same four patients’ cases as in Fig. 2. EAM surfaces were co-registered with the DT surfaces (see Methods) to facilitate direct comparison between the **Gen**DIRECT ablation targets (orange) and the actual clinical index ablation lesions (dark red) on the RV. All performance metrics are summarized in Table 1. Comparison results demonstrate a high degree of concordance between **Gen**DIRECT-predicted targets and clinical index ablation lesions, with a median sensitivity of 89.47%, specificity of 99.34% and Dice score of 89.47%. The false detection rate (FDR) was 1.41%. The average distance between the two predicted and clinical ablation point clouds was 3.93mm. These results underscore the predictive capability of **Gen**DIRECT to non-invasively and pre-procedurally determine index ablation targets for ARVC patients.

Figure 3b compares the volumes of **Gen**DIRECT predicted lesions versus those of the clinical index-ablation lesions. **Gen**DIRECT lesion sizes are significantly smaller than those of the clinical index ablation (2.70 ± 1.69 cm3 vs. 4.29 ± 2.27 cm3, ****p < 0.0001). This trend persists when analyzed by genotype. In the GE cohort (*N* = 13), the average **Gen**DIRECT-predicted lesion volume was 3.04 ± 1.63 cm^3^, which was significantly smaller than the clinical lesion volume of 4.92 ± 2.94 cm^3^ (*p* < 0.05). Similarly, for *PKP2* patients (*N* = 12), the **Gen**DIRECT-predicted lesion volume was 3.31 ± 2.43 cm^3^, significantly smaller than the clinical lesion volume of 5.22 ± 3.01 cm^3^ (*p* < 0.01). Total ablation volumes, whether identified by **Gen**DIRECT or clinical ablation, were similar between GE and *PKP2* patients. Moreover, **Gen**DIRECT targets were highly localized, precisely pinpointing the critical parts of the VT circuits. An example of this is in Fig. 3a, where the **Gen**DIRECT ablation targets of patient 14 DT are directly at the two VT isthmuses (shown in Fig. 2), while, the clinical ablation lesions, although covering approximately the same locations, were scattered over a much larger area. These results underscore the precision of **Gen**DIRECT in predicting the optimal lesions for VT termination that also minimize myocardial damage, which is an important advantage of the approach.

### GenDIRECT identifies pre-procedurally locations of emergent post-ablation VTs that are captured clinically only at redo procedures

As noted in patient demographics, in five out of the 30 patients the index ablation failed and their VT recurred, requiring re-hospitalization and a repeat procedure. Currently, there are no tools or approaches to predict whether a given ARVC patient will have VT recurrence post-ablation, or how to prevent the emergence of such VTs. Improvements in catheter design or mapping technologies cannot achieve this, as following the ablation procedure, a new substrate that consists of the remaining native fibrosis/scar plus the ablation lesions (non-conducting tissue) is created. This new substrate can be arrhythmogenic itself, giving rise to new (emergent) VTs. The **Gen**DIRECT technology is uniquely designed to predict all ablation targets at once, before the first procedure -- those inducible in the native scar/fibrosis substrate as well as those that could emerge post-ablation. Capturing all these targets pre-procedure at once eliminates the need for redo ablations and re-hospitalization.

**Fig. 4:**
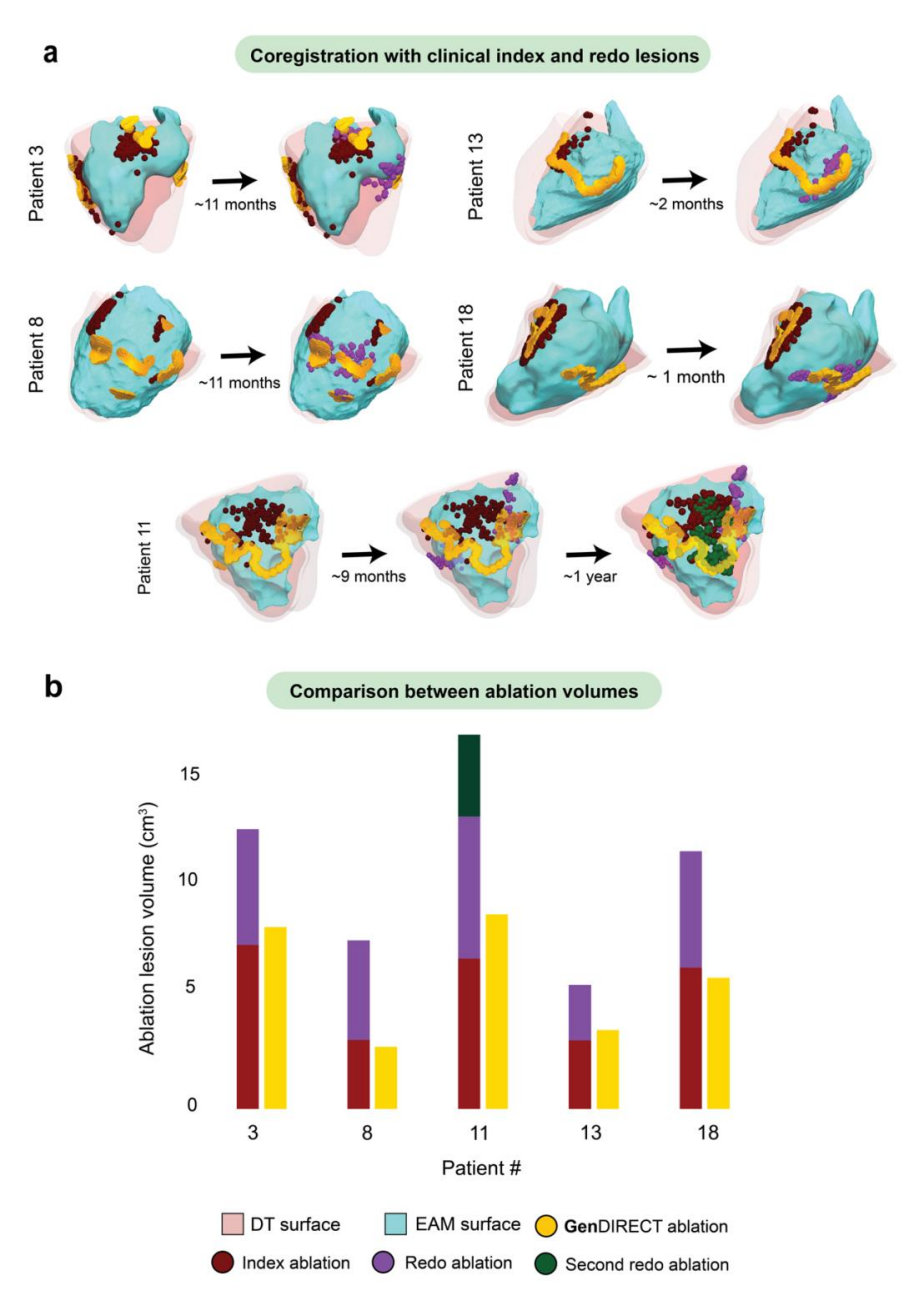
Colocalization of GenDIRECT ablation targets with clinical index and redo ablation lesions in patients who had repeated VT ablation procedures. **a** DTs of five patient cases in which VT recurred post-ablation and required redo ablation procedures. Clinical index ablation lesions (dark red), redo ablation lesions (magenta), and the second redo ablation lesions, if existing (dark green), were mapped onto the index EAM surfaces (cyan) and co-registered with the DT surfaces (light pink). The time intervals between the index and repeat procedures are indicated by the number beneath the arrows. **b** Bar plots comparing the total clinically ablated volumes (sum of index and redo procedure lesion volumes) with the **Gen**DIRECT-predicted ablation target volumes for each patient. The colors of the bar plots correspond to the same color scheme used in (**a**).

For the five patients whose VT recurred and who underwent redo ablation, we compared the clinical ablation lesions from both the index and redo procedures with the **Gen**DIRECT targets predicted pre-procedurally. As shown in Fig. 4a, the **Gen**DIRECT-predicted lesions that successfully terminated all VTs in the DTs demonstrated strong spatial concordance with the combined clinical lesions from both procedures. Quantitatively, **Gen**DIRECT predictions achieved a median sensitivity of 82.56%, specificity of 92.86%, and a Dice score of 83.33%, with a median FDR of 6.25%. The average distance between the predicted and clinical ablation point clouds was 4.09 mm. Figure 4b highlights the differences between the **Gen**DIRECT lesions volumes and the total clinical lesion volumes (sum of the index and redo lesion volumes). For each of the redo patients, **Gen**DIRECT-predicted lesions had smaller volumes, preserving cardiac tissue and thus myocardial function.

For example, in patient 3, **Gen**DIRECT predicted targets (orange) in the mid-to-base anterior, basal lateral, and mid-posterior regions, yielding an ablated volume of 7.79 cm^3^. Clinically, this patient underwent two ablations 11 months apart. The initial ablation targeted the posterior and lateral RV wall (dark red), ablating a 7.02 cm^3^ of tissue, while the redo focused on the anterior RV, particularly below the RVOT (purple), creating an additional 4.96 cm^3^ lesion. The clinical lesions from both procedures co-localized with the **Gen**DIRECT-predicted targets. Similarly, for patient 11, **Gen**DIRECT predicted a “V”-shaped target extending from the basal anterolateral to the mid-lateral and basal posterolateral regions, with a volume of 8.33 cm^3^. This patient underwent three clinical ablations: the initial procedure targeted the basal lateral RV (dark red), resulting in a lesion of 6.44 cm^3^; the first redo targeted the anterior and posterior regions (magenta), ablating 6.09 cm^3^; and the second redo targeted the mid-lateral RV wall (dark green), creating an additional lesion of 3.50 cm^3^. The clinical lesions from all three procedures colocalized with the **Gen**DIRECT targets, but with a difference of 7.70 cm^3^ total lesion volume. Should the **Gen**DIRECT prediction have been used pre-procedurally in these VT recurrence patients, its guidance would have resulted in all targets being ablated at once, at a single procedure. The power of **Gen**DIRECT is in its prediction of the “invisible” (i.e. latent) VTs that occur post-procedure (or because of it).

### Genotypic difference in the correlation between predicted ablation targets and structural substrates

As outlined in Table 2, all patients in our cohort had VTs inducible during a clinical ablation procedure. However, in clinical practice, often VT induction cannot be attempted because the patient is hemodynamically unstable. In such cases, the current clinical strategy involves ablating all regions exhibiting low-voltage potentials based on bipolar voltage mapping^40^, as the latter typically correlates with fibrosis/scar substrates^41^. However, ablation of low voltage areas may not be equally effective for the different ARVC genotypes due to the distinct underlying mechanisms of arrhythmogenesis^35^, and indiscriminate ablation can severely impair cardiac function^42^. Therefore, our study sought to explore the correlation between structural remodeling (fibrosis/scar) and ablation targets from clinical ablation and **Gen**Direct prediction in the context of the genotypic differences in ARVC patients to better inform ablation strategies if **Gen**DIRECT cannot be used to guide ablation.

**Fig. 5:**
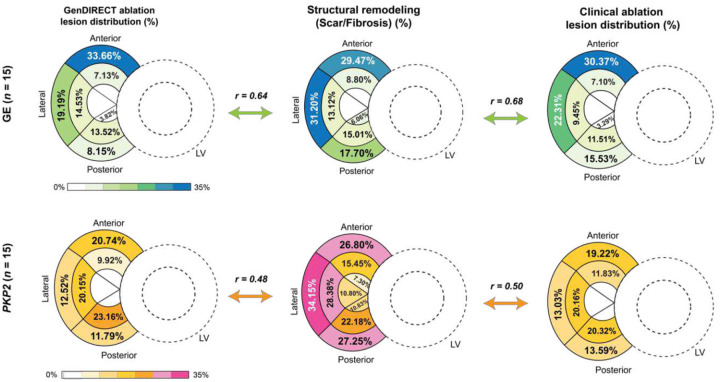
Correlation between structural remodeling (scar/fibrosis) and ablation lesion distributions. Bullseye plots depict distributions of: **Gen**DIRECT ablation targets (left), fibrosis/scar (middle), and clinical ablation lesions (right) for GE patients (top row, *N* = 15) and *PKP2* patients (bottom row, *N* = 15). For the ablation lesion distributions, the number in each AHA segment represents the average proportion of ablation targets that occur in this AHA segment. For structural remodeling, the number labeled in each AHA segment represents the average proportion of tissue identified as scar/fibrosis (based on LGE-MRI) within the segment. Pearson correlation coefficients (*r*) between ablation lesion distribution and fibrosis/scar distribution were computed for both groups and are presented between the bullseye plots. A stronger correlation between ablation targets and fibrosis/scar tissue is observed in the GE cohort compared to the *PKP2* cohort. As expected, **Gen**DIRECT and clinical ablation exhibit identical correlation coefficients between ablation lesions and fibrosis/scar distributions for both genotype groups.

For each ARVC genotype group, the average proportion of fibrosis/scar and ablation targets were computed for each AHA segment (see Methods for detailed description), as displayed in the color-scaled bullseye plots of Fig. 5. While the total volume of tissue ablated was similar between the GE and *PKP2* genotype groups, as mentioned previously, the distributions of **Gen**DIRECT targets ablation targets differed. In the GE group, 33.66% were located in the basal anterior region of the RV, which corresponded to a high fibrosis/scar density of 29.47%. However, in the *PKP2* group, high fibrosis/scar densities were found in the basal lateral and basal posterior regions (34.15% and 27.25%, respectively), but only 12.52% and 11.79% of **Gen**DIRECT ablation targets were located in these two segments. Further quantitative analysis revealed a stronger correlation between **Gen**DIRECT ablation targets and fibrosis/scar tissue in the GE cohort (*r* = 0.64, *p* < 0.0001) compared to the *PKP2* cohort (*r* = 0.48, *p* < 0.0001). A similar genotype-specific difference was also observed when comparing the correlation between clinical ablation lesions for inducible VTs and fibrosis/scar distribution (GE: *r* = 0.68, *p* < 0.0001; *PKP2*: *r* = 0.5, *p* < 0.0001).

Our findings reveal a genotypic difference in the correlation between ablation targets, identified either by **Gen**DIRECT or clinical VT induction, with structural remodeling (fibrosis/scar). The lower correlation in *PKP2* group suggests that indiscriminately ablating the low-voltage regions in *PKP2* patients with hemodynamic intolerance may have limited therapeutic efficacy and can lead to excessive lesion size.

## Discussion

The study introduces **Gen**DIRECT, a novel technology that non-invasively predicts, before the procedure, the optimal catheter ablation targets for VT in ARVC patients via personalized heart DT simulations. The technology entails the construction of heart DTs from the patients’ clinical LGE-MRI scans and genetic testing data. In this clinical and digital-twinning study in ARVC, we demonstrate how **Gen**DIRECT embodies a “test-before-treat” strategy and effectively bypasses the lengthy clinical mapping process to pinpoint the critical parts of the VT circuits (i.e. the targets for ablation). Importantly, **Gen**DIRECT is uniquely designed to predict not only the targets for elimination of all VTs inducible in the native scar/fibrosis substrate, but also those that emerge post-ablation (or because of ablation). Currently, there are no other approaches to predict whether a given ARVC patient will have VT recurrence post-ablation, or how to prevent the emergence of such VTs. Predicting all these targets at once pre-procedure and using them to guide the clinical procedure has the potential to eliminate the need for redo ablations and re-hospitalization. There is presently no technology or approach capable of predicting arrhythmogenic propensity within the post-ablation substrate and delivering this information in advance of the clinical procedure, underscoring the critical unmet need addressed by our work. With its ability to predict potential emergent VTs post-ablation, **Gen**DIRECT is poised to engender a paradigm shift in VT management by preventing repeat ablations. Overall, the clinical implementation of **Gen**DIRECT could lead to shorter procedure times, improved outcomes, reduced risks of VT recurrence, and lower healthcare costs.

Comparison of **Gen**DIRECT target lesions with the clinical ablation data in patients with inducible VT demonstrated excellent co-localization of the predicted with the clinical ablation lesions in ARVC patients undergoing index (first) ablation, regardless of the different patient genotypes (GE or *PKP2*). Similarly, our study correctly predicted, using only pre-procedural data, the post-ablation VTs that had formed many months after the procedure which necessitated the patient to undergo a redo ablation and re-hospitalization. Histological research has shown that ARVC is associated with apoptotic cell death and abnormal inflammatory immune response^45, 46^, therefore, identifying the optimal ablation targets that terminate all sustained VTs while introducing less trauma to the ARVC heart is crucial for successful patient outcomes. Our blinded comparison with clinical data, in both index and redo procedures, revealed that **Gen**DIRECT predicts significantly smaller lesion volumes than those created by clinical ablation, indicating less damage to heart tissue and better preservation of cardiac function. Importantly, research has shown that nearly 70% of patients experience VT recurrence and require multiple ablations after the index procedure in ARVC^47^.

Integrating **Gen**DIRECT into clinical ARVC ablation workflows offers several key advantages. From a procedural standpoint, **Gen**DIRECT is designed as a streamlined pre-ablation planning platform, enabling the operators to efficiently complete the planning tasks. Once determined, the **Gen**DIRECT-predicted ablation targets can be seamlessly incorporated into the clinical EAM system to guide the procedure. Indeed, our recent clinical validation studies of the DT technology have used direct import of the DT predictions into the EAM system in prospective studies^43, 44^. Moreover, these validation studies demonstrated that DT predictions reliably correspond to EP abnormalities observed in invasive clinical recordings, further reinforcing the general clinical utility of the DT technology.

**Gen**DIRECT is the first heart DT approach specifically designed to optimize ablation for a heart condition that predominantly affects the RV. While advancements in in predicting the VT circuits have been made, these efforts mostly focused on the LV^48, 49, 50^. As the LV has more than twice the thickness of the RV^51^, image processing of the LV is much simpler than that of the RV. Here, **Gen**DIRECT entails image segmentation and structural substrate identification on the RV, overcoming the unique challenges posed by RV anatomy. Furthermore, mapping the RV in a clinical setting is more challenging compared to the LV^52^, which further underscores the potential utility of **Gen**DIRECT. Its ability to predict ablation targets pre-procedurally would significantly impacts the overall efficacy and efficiency of the ablation procedure.

Our study also provides valuable insights into the genotypic differences among ARVC patients that could improve current clinical ablation strategies. We documented notable distinctions between GE and *PKP2* patients regarding the correlation between clinical ablation targets and structural remodeling, with *PKP2* patients exhibiting a weaker correlation between ablation targets and fibrotic/scarred regions. This is consistent with our previous mechanistic findings that the VTs in *PKP2* patients are not driven mainly by the structural remodeling but rather involve a large functional component driven by the PKP2-induced changes in cell and tissue EP properties^35^. This insight could substantially influence current ablation approaches when VT induction is contraindicated because of hemodynamic intolerance (substrate ablation). Our results suggest that the traditional targeting of low-voltage areas^40^ may not offer the same therapeutic effectiveness in *PKP2* patients as it does in GE patients.

Looking ahead, **Gen**DIRECT offers a flexible framework that can be extended to the planning of other emergent treatments for ARVC, such as gene therapy^53, 54^. The approach could simulate the delivery of gene therapy using adeno-associated virus vectors to target regions of arrhythmia perpetration, enabling pre-injection testing of outcomes. As a versatile, multiscale computational tool, **Gen**DIRECT could integrate fundamental findings from the cellular to the tissue level and evaluate their therapeutic efficacy at the organ scale within clinically relevant scenarios. This versatility not only supports broader application in other genetic heart diseases but also sets a foundation for investigating more nuanced genotype-phenotype relationships in ARVC. In particular, as mutation-specific electrophysiological data become available, future studies could explore how different mutation types within the same ARVC genotype contribute to arrhythmogenic remodeling. Incorporating such detailed information, which would be straightforward in our **Gen**DIRECT platform, could further refine the predictive power of genotype-informed DTs and enhance the personalization of treatments.

Our study has several limitations. First, the EP properties of diffuse fibrosis in ARVC were inferred from experimental data for non-ischemic cardiomyopathy due to a lack of specific experimental studies on ARVC’s fibrosis^55^. Although our previous studies have validated the use of this fibrosis cell model in ARVC and other non-ischemic heart diseases, such as sarcoidosis, tetralogy of Fallot, and hypertrophic cardiomyopathy^30, 31, 32, 35^, future research should aim to incorporate ARVC-specific fibrotic EP properties once experimental data becomes available. Second, the current heart DTs were from patients with RV-dominant ARVC. While the DTs are perfectly capable of accommodating LV involvement, the application of **Gen**DIRECT to such a cohort has not been clinically validated due to the scarcity of clinical data on patients who have an LV-dominant genotype such as desmoplakin (*DSP*) deficiency. Third, this is a retrospective study. To demonstrate the clinical utility of **Gen**DIRECT in guiding ablation in ARVC patients, a randomized clinical trial with a larger, prospectively enrolled cohort will be needed.

In conclusion, the study presents **Gen**DIRECT, a genotype-specific heart DT technology designed for optimal ablation target prediction in ARVC prior to the clinical procedure. We successfully tested the feasibility of **Gen**DIRECT via blinded comparison with clinical ablation data, demonstrating its ability to effectively terminate inducible VTs and prevent the emergence of new post-ablation arrhythmias. Our findings highlight its potential to streamline clinical VT ablation workflows by improving procedural efficiency and delivering precise, effective treatment that improves patient outcomes. As a powerful tool, we envision **Gen**DIRECT not only advancing individualized ablation strategies for ARVC but also contributing to the development of innovative therapeutic approaches for broader clinical applications.

## Methods

### Study design and participants

**Fig 6:**
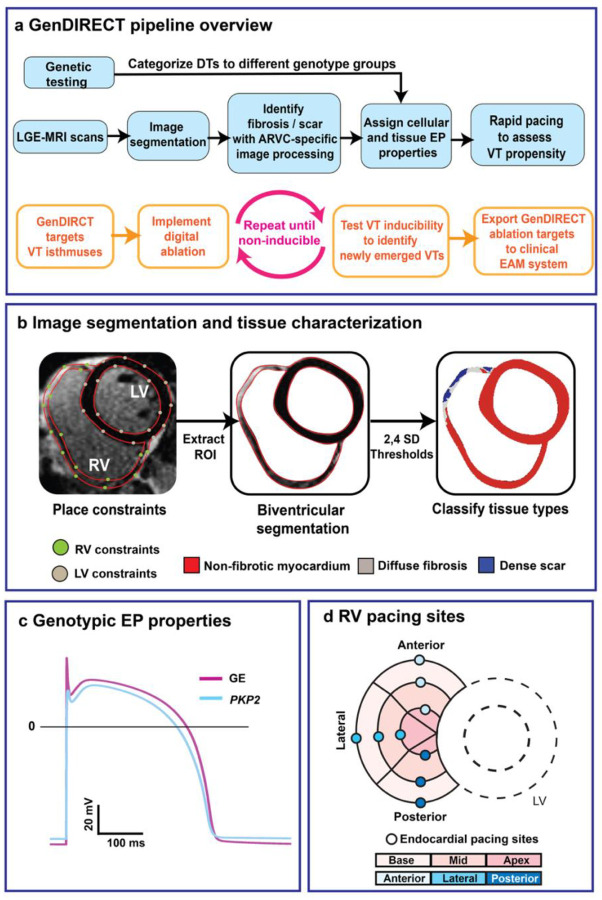
Overview of the GenDIRECT pipeline. **a** Flowchart outlining the step-by-step reconstruction of heart DTs and VT assessment in patients with ARVC. LGE-MRI scans were segmented to reconstruct ventricular geometry and identify structural substrates such as fibrosis and scar in RV. Patient genetic testing results were used to assign cell and tissue EP properties. The DTs were subjected to rapid pacing to induce VTs. The orange flowchart details the **Gen**DIRECT-guided ablation target prediction, where the process is iterated until the DT becomes non-inducible for VT from any of the 9 RV pacing locations. When implemented in the clinical settings, these predicted ablation targets would be imported to the clinical EAM system for real-time guidance. **b** Semi-automatic ARVC-specific image processing was used to classify voxels within the segmented contours into three distinct tissue types. **c** Comparison of action potentials for the GE and *PKP2* cell models. **d** Nine uniformly distributed pacing locations on the RV endocardial surface used in VT induction.

In this retrospective clinical and digital-twinning application termed **Gen**DIRECT, 30 patients were selected based on available data that met our inclusion and exclusion criteria. The study design overview was illustrated in Fig. 1. For each patient, a personalized genotype-specific DT was developed using the methodological pipeline outlined in Fig. 6. Here we present the workflow overview for the **Gen**DIRECT approach, as shown in Fig. 6a. Briefly, geometrical representations of the ventricles (RV and LV) were generated for each patient, incorporating personalized fibrosis and scar distributions. Each DT was constructed by populating the imaging-based geometrical model of the patient’s heart with the genotype-specific cellular and tissue EP properties. VT circuits were then induced in the DTs through a validated rapid pacing protocol, applied from nine RV endocardial surface locations. In total, 270 simulation tasks (30 patients × 9 pacing sites per patient) were analyzed to identify critical sites of induced VT reentrant circuits as the targets for **Gen**DIRECT ablation. VT inducibility tests were conducted after each ablation in the DT. Optimal ablation targets that successfully terminated all induced VT circuits (native or emergent) in the DTs were compared with clinical ablation lesions obtained from clinical EAM data. To ensure unbiased segmentation and model reconstruction, simulation operators were blinded to clinical ablation records.

Patients were recruited from the Johns Hopkins ARVC registry, and the study received approval from the institutional review board. Inclusion criteria were as follows: patients need to have (1) a definitive diagnosis of ARVC according to the 2010 Task Force Criteria^56^; (2) history of undergoing VT ablation procedures with inducibility during the procedure; (3) availability of comprehensive clinical ablation data including EP report, EAM data, and lesion information; (4) presence of RV fibrotic remodeling on LGE-MRI scans; and (5) completion of genetic testing for ARVC risk variants, with results falling into either gene-elusive (GE) or the plakophilin-2 loss-of-function pathogenic variant (*PKP2*). Exclusion criteria included (1) poor image quality of LGE-MRI scans that precluded segmentation; (2) absence of sustained VT inducibility during clinical ablation; (3) missing of ablation location data (i.e. lesion points missing). These criteria were determined prior to searching the Johns Hopkins ARVC database. Ultimately, a total of 38 patients met the inclusion criteria for the study, of which 8 were excluded based on exclusion criteria.

Of the 30 included patients, 22 underwent LGE-MRI at the Johns Hopkins Radiology Department. Scans were acquired on 1.5-T scanners (Avanto, Siemens, Erlangen, Germany or GE Medical Systems, Waukesha, WI), following intravenous injection of gadolinium of 0.2mmol/kg. The remaining eight patient had their LGE-MRI performed at outside institutions before referral to the Johns Hopkins ARVC Center for consultation; scanner details were unavailable to the authors. Across all patients, LGE-MRI scans had a median of 12 slices each, an average slice thickness of 9.33 ± 1.92 mm, and an average in-plane axial resolution of 1.50 ± 0.38 mm. For patients with ICD implanted before LGE-MRI scans were taken, wideband LGE-MRI sequences were used to reduce artifact. All the images were clinically reviewed to determine the presence/absence of RV enhancement and to ensure consistent image quality for digital twin reconstruction (S.Z.).

### Quantitative analysis of EAM surface characteristics

The surface area of each patient’s clinical Electroanatomic Map (EAM) was calculated using the surface integral functionality within Paraview (www.paraview.org, Kitware, 2015). To assess the mapping point density, a circular region with a 1 cm radius was defined, centered sequentially at each point on the EAM surface. For each of these defined regions, the number of neighboring EAM points falling within the 1 cm radius was counted. The mapping point density for the EAM was then determined by averaging these counts across all analyzed points on the surface.

### GenDIRECT approach specifics

#### Heart DT geometrical reconstruction

Three-dimensional DTs were reconstructed from 2D LGE-MRI scans, as detailed in Fig. 6b. Clinical scans were first resampled into an isotropic resolution of 0.35 × 0.35 × 0.35 mm using the open-source software 3D slicer^57^. Biventricular segmentation was performed using the semi-automatic segmentation method (CardioViz3D) described in our previous studies^30, 32, 35, 48^, where both endocardial and epicardial surfaces were defined with contour points to delineate the myocardial region. Otsu thresholding was then applied to binarize the myocardium into high- and low-intensity regions. Due to the disease characteristics of this cohort of ARVC patients, the DTs focused exclusively on RV structural remodeling, as the left ventricle was non-fibrotic. The mean of the lower LGE-MRI signal intensity region on the RV was used as the reference mean for non-fibrotic myocardium. A threshold of ≥ 2 standard deviations (SD) but < 4 SD was used to categorize diffuse fibrosis, and a threshold of ≥ 4 was used for dense scar.

Following the geometrical reconstruction of all DTs (shown in Fig. S1), we quantified the structural remodeling distributions in the GE and *PKP2* groups, which were used for correlation analyses presented in Fig. 5. The structural remodeling in ARVC patients comprises diffuse fibrosis and dense scar. For each AHA segment of the RV in each patient’s DT, the regional proportion of scar/fibrosis was calculated by dividing the scar/fibrosis volume by the corresponding AHA segment volume, yielding a percentage value for each segment. The regional densities across all AHA segments were then averaged within each genotype group to represent the genotypic distribution of scar/fibrosis.

#### Computational ventricular meshes and fiber orientations

Prior to conducting simulations, 3D finite-element tetrahedral meshes with a mean edge length of 400μm and an average number of 5.1 million nodes were generated (Mimics Innovation Suite; Materialise, Leuven, Belgium) for the reconstructed ventricular geometries. We chose the finite element size based on the need to resolve wavefront propagation in the simulations while minimizing computational expense to ensure the computational load can accommodate the clinical workflow timeline. Fiber orientations were assigned to each mesh element using a validated rule-based method^58^ used in a number of our previous studies^30, 32, 36, 59^. In brief, at each point within the DT, the Laplace-Dirichlet method was used to determine transmural and apicobasal directions. Subsequently, bidirectional spherical linear interpolation was used to establish fiber orientations based on the fiber orientation rules.

#### Genotype-specific EP properties

Our previous research has demonstrated the importance of incorporating ARVC genotype-specific EP properties into ARVC DTs to accurately characterize ventricular arrhythmogenesis^35^. Accordingly, in our **Gen**DIRECT approach, the EP properties of non-fibrotic myocardium were modeled using distinct cell models for GE and *PKP2* patients, the two genotypes represented in our patient cohort. Detailed descriptions of these ion channel modifications, as well as the cellular model development and validation, were provided in our previous study^35^. In brief, we used the 2006 tenTusscher and Panfilov (TT2) human ventricular model, supplemented with a late sodium current formulation^60^ as the GE cell model. We then modified this baseline model to reflect the pathological ionic remodeling caused by *PKP2* loss-of-function mutations, based on experimental findings^61^.

As shown in Fig. 6c, these channel adjustments resulted in a *PKP2* action potential (AP) with a slower upstroke velocity, lower peak, and more positive resting membrane potential than the baseline cell AP. Consequently, the non-fibrotic myocardium in the *PKP2* DTs had slower conduction velocity and a flattened restitution curve, indicating poorer AP duration adaptation to rapid pacing. For regions of diffuse fibrosis, the differences in EP properties between GE and *PKP2* are poorly understood. Therefore, we chose a previously validated version of the TT2 model with channel modifications based on experimental data of non-ischemic cardiomyopathy^30, 32, 55^. This diffuse fibrosis cell model exhibited a longer AP duration and slower conduction velocity than both GE and *PKP2* models.

Conduction velocity (CV) and conductivity values for non-fibrotic and fibrotic regions, in both longitudinal and transverse fiber directions for the GE and *PKP2* genotypes are summarized in Table. S1. The CV for GE non-fibrotic tissue was consistent with our previous research in non-ischemic cardiomyopathy^31, 32^. In PKP2 non-fibrotic tissue, longitudinal and transverse CVs were reduced by 60% and 48%, respectively, compared to those in GE non-fibrotic tissue. These reductions were reflected in our digital twins and agree with previously reported data in the literature^61, 62^. CVs in fibrotic regions were scaled down in both directions, in line with our earlier studies^30, 31, 32^. Logarithmic fitting was performed between paired conductivity values and CVs for each tissue type, and conductivity values at a specific target CVs were derived using the relationship found. Dense scar tissue was modeled as electrical insulator.

#### Identifying VT circuits in DTs

VT reentrant circuits were induced in each patient’s DT using a previously validated rapid-pacing protocol^48, 59, 63^. The RV was divided into nine AHA segments (basal, middle, apical, anterior, lateral, and inferior), and pacing sites were automatically selected using the algorithm described in our prior work^35^. This sequential pacing protocol involved six stimuli (S1) at a 600ms cycle length, followed by a premature stimulus (S2) given 250ms after S1. If reentry was not induced, the S1-S2 interval was progressively reduced in 10ms increments. If arrhythmia fails to occur, additional premature stimuli (S3, S4, and S5) were administered. This pacing protocol mimics the one performed clinically. Pacing was delivered from nine equally distributed RV endocardial sites, with each AHA segment having one pacing location (Fig. 6d). Each stimulus was applied to a 1mm^3^ volume of cardiac tissue. A sustained reentry lasting two cycles at a same critical site was defined as an induced VT^48, 59^. All simulations were performed using openCARP^64^.

#### Determining critical sites of VT circuits

After inducing the VT circuits, we visualized their 3D activation maps in Meshalyzer (https://opencarp.org/documentation/examples/visualization/meshalyzer). The activation sequence was divided into eight uniformly spaced time windows, (isochrones). These isochrones identified the key components of each VT circuit: the exit, entrance, outer loop, isthmus and common pathway^36^. The green and cyan isochrones denote the critical VT isthmus, which was a primary ablation target for the **Gen**DIRECT approach.

#### GenDIRECT optimal target selection

After inducing all VT circuits and identifying their isthmuses, we defined the digital ablation lesion of each heart digital twin as the minimum volume of tissue needed be rendered electrically inert in order to terminate all inducible VT circuits. From a finite element modeling perspective, a digital lesion was created by selecting a node and all neighboring nodes within a 3,500-μm radius and assigning them insulating EP properties. To determine the minimal lesion size, the critical isthmus of the VT circuit was first chosen as the primary target of the digital lesion. Incremental additions to the digital lesion (other critical VT sites) were performed to terminate any meandering VT circuits. Line connections between adjacent ablation targets were applied when iatrogenic VTs occurred with a pathway between the two lesions. Following each round of digital ablation, the rapid pacing protocol was repeated to the post-ablated DT to determine whether new VTs emerged. If so, they were digitally ablated using the same methodology, and this process was repeated until no further post-ablation VTs could be induced.

### Comparing GenDIRECT-guided ablations with clinical ablation data

The ablation targets predicted by **Gen**DIRECT were compared with clinical ablation lesions derived from the ablation catheter tip locations acquired during the procedure.

#### DT-to-EAM registration

Among the 30 patients in the cohort, 20 underwent EAM using the EnSite Precision (Abbott) system, while the remaining 10 underwent EAM using the CARTO 3 (BioSense Webster) system. The workflow for the registration of the **Gen**DIRECT predictions and the clinical EAM data is illustrated in Fig. S2. As shown in Fig. S2a, each EAM dataset had anatomical landmarks identified from pre-procedural CT scans which were registered to the EAM surface during the ablation procedure. First, these landmarks (the CT surfaces) were co-registered to the DT’s landmarks (the DT’s surfaces) by utilizing a fiducial registration method embedded in Paraview (www.paraview.org, Kitware, 2015) resulting in a registration transformation matrix. This transformation matrix was then applied to co-register the EAM surface and ablation catheter tip locations to the DT. Using the same method, in the five cases where redo ablations were performed, the redo EAM data was first registered with the index EAM data, and the index EAM data was then registered with the DT’s endocardial surface. Ultimately, the combined index and redo ablation lesions were projected onto the DT.

#### Ablation lesion size quantification

As shown in Fig. S2b, following the DT-to-EAM registration, catheter tip points were projected onto the closest point on the RV endocardial surface of the DT. Digital ablation points were similarly projected to the RV endocardial surface using the same approach. To quantify both the clinical and digital lesion volume, the lesions created by each ablation point was estimated as a semi-spherical volume with a radius of 3,500-μm. The rationale of this estimation is based on the standard ablation catheter tip size of 3,500 μm^65^. We utilized the open-source software Paraview (www.paraview.org, Kitware, 2015) to calculate the volume integral of the ablation lesions was, which avoided double-counting overlapped regions.

### Statistical information

In Figs. 3 and 4, the Dice Similarity Coefficient (Dice score) was calculated to assess the colocalization between **Gen**DIRECT ablation targets and clinical ablation lesions. This metric quantifies the degree of overlap between two datasets, with a score ranging from 0 (no overlap) to 1 (complete overlap). The Wilcoxon paired signed-rank test was used to compare the volumes of **Gen**DIRECT ablation targets with clinical ablation lesions across the entire cohort. In Fig. 5, the Pearson correlation coefficient was used to quantify the correlation between the structural substrate (scar/fibrosis) and **Gen**DIRECT ablation targets.

## Supplementary Material

Supplementary Files

This is a list of supplementary files associated with this preprint. Click to download.

- manuscriptCVRsubmissionsupp.pdf

## Figures and Tables

**Table 1:** Evaluation of GenDIRECT performance in predicting ablation targets compared with clinical ablation targets for patients with only index ablation (*N* = 25) and patients with both index and redo ablations (*N* = 5).

Metrics	Index ablation (*N* = 25)	Redo ablation (*N* = 5)
Sensitivity (%)	89.47 [73.04 – 96.23]	82.56 [80.01 – 91.19]
Specificity (%)	99.43 [86.71 – 1.00]	92.86 [85.63 – 94.73]
FDR (%)	1.41 [0.00 – 10.65]	6.25 [3.62 – 16.00]
Dice score (%)	89.47 [73.04 – 99.23]	83.33 [79.86 – 84.41]
Average distance (mm)	3.93 [3.21 – 5.02]	4.09 [3.89 – 4.94]

Data are presented as median [IQR], where the interquartile range (IQR) corresponds to the 25th to 75th percentile (Q1–Q3). FDR: false detection rate.

**Table 2:** Patient characteristics.

Patient characteristics (*N* = 30)	
Age, y	41.5 [19–73]
Male	12 (40)
Confirmed ARVC diagnosis	30 (100)
Genotype (*PKP2*)	15 (50)
Anti-arrhythmic drug	26 (87)
*Class IC*	*23*
*Class III*	*3*
*Both class IC and III*	*4*
RV LGE hyperenhancement	30 (100)
ICD before LGE-MRI	11 (37)
Ablation characteristics	
Age of first ablation, y	34 [17–69]
Ablation year	2019 [2012–2022]
EAM system (EnSite)	20
Redo ablation	5
ICD before ablation	24 (80)

* Confirmed ARVC diagnosis is based on *2010 Task Force Criteria* Continuous variables are given as median [range]. Categorical variables are expressed as the count (percentage). ICD – implanted cardioverter-defibrillator; y – year; EAM – Electroanatomic mapping; EnSite – EnSite precision cardiac mapping system (Abbott).

**Table 3:** **Ablation technique summary**.

Ablation technique summary (*N* = 30)		
VT induction		30 (100)
Pace mapping		28 (93)
Substrate-based lesions		27 (90)
Fractionated/late potentials		25 (83)
Slow conduction zones		2 (7)
Patients without VT recurrence (*N* = 25)		
Epicardial mapping		25 (100)
Epicardial ablation		24 (96)
Endocardial mapping		19 (76)
Endocardial ablation		10 (40)
Patients with VT recurrence (*N* = 5)		
**Index ablation**	Epicardial mapping	5 (100)
	Epicardial ablation	5 (100)
	Endocardial mapping	5 (100)
	Endocardial ablation	3 (60)
**Redo ablation**	Epicardial mapping	5 (100)
	Epicardial ablation	5 (100)
	Endocardial mapping	3 (60)
	Endocardial ablation	3 (60)

Categorical variables are expressed as the count (percentage).

## Data Availability

Patient-derived data including CMR images are not publicly available to respect patient privacy. Interested parties wishing to obtain these data for non-commercial reuse should contact the corresponding author via email; the request will need to be approved by IRB.

## Code Availability

The image processing software CardioViz3D can be freely obtained from http://www-sop.inria.fr/asclepios/software/CardioViz3D. The cell models are freely available from the repository CellML (https://www.cellml.org). Documentation and instructions on the use of the openCARP cardiac electrophysiology simulator and meshalyer visualization software are available via https://opencarp.org/.
